# From pills to patients: an evaluation of data sources to determine the number of people living with HIV who are receiving antiretroviral therapy in Germany

**DOI:** 10.1186/s12889-015-1598-4

**Published:** 2015-03-17

**Authors:** Daniel Schmidt, Christian Kollan, Matthias Stoll, Hans-Jürgen Stellbrink, Andreas Plettenberg, Gerd Fätkenheuer, Frank Bergmann, Johannes R Bogner, Jan van Lunzen, Jürgen Rockstroh, Stefan Esser, Björn-Erik Ole Jensen, Heinz-August Horst, Carlos Fritzsche, Andrea Kühne, Matthias an der Heiden, Osamah Hamouda, Barbara Bartmeyer, ClinSurv Study Group

**Affiliations:** Robert Koch Institute, Department of Infectious Disease Epidemiology, HIV/AIDS, STI and Blood-borne Infections, Berlin, Germany; Clinic for Immunology and Rheumatology, Infectious Diseases Unit, Medical University Hannover, Hannover, Germany; ICH Study Centre Hamburg, Hamburg, Germany; Ifi-Institute for Interdisciplinary Medicine, Hamburg, Germany; Clinic of Internal Medicine, University Köln, Köln, Germany; Department of Infectious Diseases and Pulmonary Medicine, Charité University Medicine Berlin, Berlin, Germany; Department of Infectious Disease, Med IV, University Hospital of Munich, Munich, Germany; Section Infectiology, University Medical Center Hamburg-Eppendorf, Hamburg, Germany; Department of Internal Medicine, University of Bonn, Bonn, Germany; Clinic for Dermatology, Infectious Diseases, University Hospital Essen, Essen, Germany; Department of Gastroenterology, Hepatology and Infectious Diseases, Heinrich Heine University Düsseldorf, Düsseldorf, Germany; Medical Clinic, University Schleswig Holstein, Campus Kiel, Germany; University Hospital Rostock, Rostock, Germany

**Keywords:** HIV treatment, Composition of ART regimen, Antiretroviral drug classes, Health market research

## Abstract

**Background:**

This study aimed to determine the number of people living with HIV receiving antiretroviral therapy (ART) between 2006 and 2013 in Germany by using the available numbers of antiretroviral drug prescriptions and treatment data from the ClinSurv HIV cohort (CSH).

**Methods:**

The CSH is a multi-centre, open, long-term observational cohort study with an average number of 10.400 patients in the study period 2006–2013. ART has been documented on average for 86% of those CSH patients and medication history is well documented in the CSH.

The antiretroviral prescription data (APD) are reported by billing centres for pharmacies covering >99% of nationwide pharmacy sales of all individuals with statutory health insurance (SHI) in Germany (~85%). Exactly one thiacytidine-containing medication (TCM) with either emtricitabine or lamivudine is present in all antiretroviral fixed-dose combinations (FDCs). Thus, each daily dose of TCM documented in the APD is presumed to be representative of one person per day receiving ART. The proportion of non-TCM regimen days in the CSH was used to determine the corresponding number of individuals in the APD.

**Results:**

The proportion of CSH patients receiving TCMs increased continuously over time (from 85% to 93%; 2006–2013). In contrast, treatment interruptions declined remarkably (from 11% to 2%; 2006–2013). The total number of HIV-infected people with ART experience in Germany increased from 31,500 (95% CI 31,000-32,000) individuals to 54,000 (95% CI 53,000-55,500) over the observation period (including 16.3% without SHI and persons who had interrupted ART). An average increase of approximately 2,900 persons receiving ART was observed annually in Germany.

**Conclusions:**

A substantial increase in the number of people receiving ART was observed from 2006 to 2013 in Germany.

Currently, the majority (93%) of antiretroviral regimens in the CSH included TCMs with ongoing use of FDCs. Based on these results, the future number of people receiving ART could be estimated by exclusively using TCM prescriptions, assuming that treatment guidelines will not change with respect to TCM use in ART regimens.

## Background

Combined antiretroviral therapy (ART) as a standard of care has dramatically reduced mortality and morbidity and has led to an enormous increase in quality of life among people infected with HIV [1,2]. In most patients who receive ART, progression to AIDS or death is increasingly rare [3-5], and their life expectancies have significantly improved [6-8]. However, ART is a complex and lifelong therapy that must be well monitored, coordinated and tracked. Although ART is still not available for a large number of people in need, especially in developing countries [9], the number of people living with HIV who are receiving treatment is increasing worldwide [9]. In industrialised countries, a large number of people living with HIV are under treatment [10]. As HIV has become a chronic disease, an increasing number of people must be treated for decades, making it an important economic and public health issue to gain information on this group. Information on the current number of people living with HIV receiving ART in Germany is scarce owing to a lack of data, and access to personal-level drug prescription data is forbidden because of data protection.

HIV treatment in Germany is characterized by a decentralised structure. Medical care is mainly provided by specialized outpatient centres and office-based HIV specialists, and unlike in many countries people can consult a doctor of their own choice at any time and anywhere in the country. Furthermore, health care in Germany is compulsory for all German citizens and legal residents and is mostly provided by statutory health insurance (SHI) or private health insurance (PHI) [11-13]. SHI occupies a central position in the German health care system. Approximately 85% of German residents are covered by SHI, and nearly 60% of the total health expenditures are borne by SHI [12]. SHI reimburses pharmacies for the prescriptions of those who are covered via specialised pharmacy billing centres. Therefore, the prescription details are electronically recorded. The recording and use of these data are regulated by the social security law (§300 SGB V). Data from health services research such as electronically recorded pharmacy data are being increasingly used for research in Germany. Nevertheless, public health studies using data representing nearly all persons covered by SHI are scarce.

The prescription data include all antiretroviral drugs, regardless of whether they are for permanent or short-term therapies, e.g., post-exposure prophylaxis (PEP). No individual information and, therefore no indications, are available. In contrast, the prospective multi-centre observational German ClinSurv HIV cohort (CSH) ongoing since 1999 is the largest available nationwide source of people infected with HIV and collects detailed information on the initiation, composition and discontinuation of individuals’ daily ART from their participating centres [14].

Since the approval of the first antiretroviral agent, at least in the industrialised world, more than 30 antiretroviral pharmaceuticals, either single-drug formulations or fixed-dose combinations, are available for the treatment of HIV infection [15]. Nucleoside/nucleotide reverse transcriptase inhibitors (NRTIs) are still the main components of antiretroviral drug combinations [16] and are recommended as an element of any first-line antiretroviral regimen by therapy guidelines [17-19]. Currently, a combination of three antiretroviral drug classes consisting of two NRTIs and a third agent, either a protease inhibitor (PI) or a non-nucleoside reverse transcriptase inhibitor (NNRTI) or an integrase inhibitor (INI), is recommended for first-line therapy [17,18]. During the last decade, it has been recommended that all first-line NRTI combinations contain an element of a thiacytidine medication (TCM), either lamivudine (3TC) or emtricitabine (FTC) [17,19,20]. The two medications are interchangeable, but because of their high antiretroviral similarity with no additional effects, concomitant use should be avoided [17]. NRTI-free regimen such as PI monotherapy are not recommended because of inferior antiviral potency [17,18,21-23]. Because standard ART consists of a combination of at least three antiretroviral drugs given in a multitude of combination regimens, it is impossible to estimate the number of people receiving ART prescriptions based on all single drugs [24]. However, virtually all ART regimens prescribed in different studies in a setting of daily clinical practice contain exactly one TCM [25-33]. Thus, each daily TCM documented in the APD may be assumed to be representative of one person per day treated with ART. It was hypothesised that the ART regimens and treatment interruptions recorded in the CSH were representative of people living with HIV under antiretroviral treatment in Germany and that the prescriptions covered by SHI were comparable with those that were not.

This study used available prescription data sources from both pharmacy billing centres and the CSH to determine the number of people living with HIV currently receiving ART, the number of HIV-infected people with ART experience, and the differences in those numbers over time between 2006 and 2013.

## Methods

### Data sources used for analysis

#### ART prescription data (APD)

ART prescription data were provided by Insight Health™ for the years 2006–2013. The data were collected on a monthly basis from billing centres that processed all reimbursed prescriptions from pharmacies based on the date of redemption at the counter. The provider claimed a coverage of >99% within the SHI prescription market. The recorded numbers of prescribed standard units (i.e., numbers of tablets) of each antiretroviral drug were used for this study.

Defined daily doses (DDDs) were determined as recommended in the treatment guidelines [17]. The number of prescribed DDDs was calculated for TCMs depending on the doses of standard units. According to our approach, a DDD that included a TCM represented one person-day, assuming that one person was treated with TCM continuously every day for a quarter, as is recommended by treatment guidelines. In the case of the prescription of a 150 mg dose of lamivudine, 2 tablets were equivalent to one DDD.

#### The German ClinSurv HIV cohort (CSH)

The Clinical Surveillance of HIV Disease is a nationwide multi-centre, open, long-term observational cohort study for the clinical surveillance of HIV in Germany. The CSH was initiated in 1999 as collaboration between major HIV treatment centres and the Robert Koch Institute (RKI) which serves as the coordinating institution. Anonymised data on patient demographics, detailed information on antiretroviral treatment, laboratory parameters and clinical events are collected biannually in a standardised format. The study design is described in detail elsewhere [14]. In the study period 2006–2013, an average number of 10.400 patients were observed and consecutively monitored at 15 clinical centres in various, predominantly urban areas in Germany. Antiretroviral treatment history, including any interruptions in treatment, is documented in detail in the CSH [14,24]. Treatment duration is calculated individually according to the beginning and end dates of each antiretroviral drug treatment. All ART documentation is assessed manually. Quality control algorithms are applied, and in the case of inconsistencies, the centres are requested to submit the revised data to the RKI [14].

The Robert Koch Institute is the German national public health institute, therefore the Federal Commissioner for Data Protection is the responsible entity for studies which are conducted by the Robert Koch Institute. Information on HIV infection collected in ClinSurv corresponds to the data reported to the RKI according to legal requirements implemented by the national Protection against Infection act (IfSG) of 2001. All patient data collected in ClinSurv are generated during routine care. The German Federal Commissioner for Data Protection therefore waived the need for ethical approval for the ClinSurv study. No written informed consent is required from patients.

The overall person-days observed from persons receiving any antiretroviral treatment between 2006 and 2013 in the CSH were analysed and categorised into three groups: medications that contained approved drugs, medications that contained at least one non-approved drug, and interrupted therapy. In the first group, we distinguished between regimens that did include a TCM and those without TCM. The numbers of all of these groups were calculated quarterly. Treatment interruption was defined as any observation time between therapy initiation and latest observed event with documented treatment discontinuation.

For the analysis of ART regimen in the CSH we separated mainly used regimens and minor regimens. Mainly used regimens were either defined as ART regimen containing two or three NRTIs and another drug class (NNRTIs, PIs, INIs) or two or three NRTIs exclusively. Minor regimens were those including more than three NRTIs and NRTI-free regimen.

### Combination of data sources

#### Determining the number of people living with HIV receiving ART

**Figure 1:**
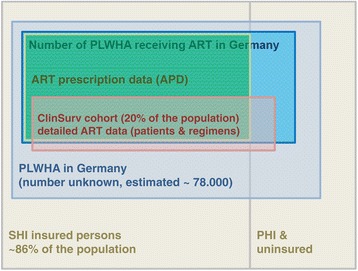
**Schematic overview of subpopulations and available data sources in Germany.** Approximately 85% of the population in Germany is covered by statutory health insurance (SHI), most of the remainder are covered by private health insurance (PHI) and a small proportion are uninsured (exact number unknown). For persons covered by the SHI, antiretroviral prescriptions are recorded and reported through antiretroviral prescription data (APD). The German ClinSurv HIV cohort (CSH) contains detailed ART history data on approximately 20% of people living with HIV in Germany receiving ART, both those who are covered by SHI and those who are not. This schematic is not to scale.

**Figure 2:**
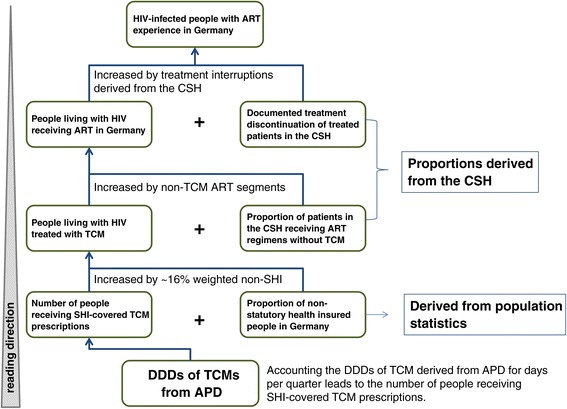
**Process diagram of used data sources and calculation steps proceeded.**

The number of prescribed DDDs of TCMs derived from ART prescription data was used to determine the number of people living with HIV receiving quarterly SHI-covered TCM containing ART in Germany. The proportion of persons covered by SHI was calculated for each federal state based on the number of persons with SHI and the population number of the respective state. To account for patients without SHI (including those privately insured, uninsured, or receiving free medical care) whose prescriptions were not covered in the APD, the number of patients was raised in average by a weighted factor of 16.3% [34]. By adding the numbers of person-days of non-TCM ART segments derived from the CSH, we determined the total number of people living with HIV receiving quarterly ART in Germany. In addition, considering the proportion of person-days with treatment interruption seen in the CSH yielded the number of patients in Germany with ART experience. For an overview of the investigated data sources, see Figures 1 and 2.

The estimated number of HIV-infected persons with ART experience was smoothed using a negative binomial regression with quadratic time trend in the period of 2006 to 2013. The statistical errors of these numbers were assumed to be independent. The independent variables considered in the negative binomial regression were the time - measured in quarters since the first quarter in year 2006 - and the square of this time. The latter variable allowed us to adjust for a slowing down of the exponentially increasing trend in the recent years.

## Results

### ClinSurv HIV cohort (CSH)

The proportion of person-days with TCM-containing regimens reported in the CSH increased continuously over the study period, from 85% in 2006/I to 93% in 2013/IV. In contrast, the proportion of person-days with any observed treatment interruption declined from 11% in 2006/I to 2% in 2013/IV. The proportion of person-days with an antiretroviral regimen that contained non-approved drugs decreased from 6% in 2006/I to 2% in 2013/IV (Table 1).

**Table 1:** **The German ClinSurv HIV cohort in the study period 2006–2013**

**Year/quarter**	**Patients under observation**	**Patients under ART time**	**Observation time**	**Time under ART or interruption**	**ART status unknown**	**Art naive**	**ART regimens with approved drugs exclusively**	**ART regimens containing non-approved drugs**	**Treatment interruptions**	**ART experienced**	**TCMs in the CSH**	**Proportion of interruptions**
	**N**		**Days**									
**2006/I**	8717	6986	753553	613673	8211	131728	516827	29909	66937	81.4%	84.5%	10.9%
**2006/II**	8856	7104	773115	630625	7907	134629	533732	32431	64462	81.6%	85.3%	10.2%
**2006/III**	9002	7214	792169	646716	7742	137766	547485	36102	63129	81.6%	86.2%	9.8%
**2006/IV**	9075	7281	803312	655415	7530	140415	558719	36453	60243	81.6%	87.0%	9.2%
**2007/I**	9267	7434	798257	652832	7219	138281	560733	33462	58637	81.8%	87.7%	9.0%
**2007/II**	9407	7552	820040	671081	7345	141682	579930	33382	57769	81.8%	88.4%	8.6%
**2007/III**	9564	7689	844690	690514	7304	146945	595635	38313	56566	81.7%	89.1%	8.2%
**2007/IV**	9683	7828	855138	704369	6948	143877	609239	39403	55727	82.4%	89.5%	7.9%
**2008/I**	9758	7937	853480	705518	6344	141676	619880	31297	54341	82.7%	89.8%	7.7%
**2008/II**	9903	8069	863850	716324	5985	141595	632750	31325	52249	82.9%	90.4%	7.3%
**2008/III**	10031	8206	884551	737289	5895	141423	656479	28383	52427	83.4%	90.7%	7.1%
**2008/IV**	10124	8340	896771	752357	5982	138495	678973	22195	51189	83.9%	91.0%	6.8%
**2009/I**	10222	8484	886182	747140	5303	133792	677700	21820	47620	84.3%	91.3%	6.4%
**2009/II**	10384	8624	910943	769502	4859	136659	700526	21972	47004	84.5%	91.6%	6.1%
**2009/III**	10569	8814	934456	791390	4779	138362	722970	22322	46098	84.7%	91.8%	5.8%
**2009/IV**	10697	8989	946177	808947	4660	132635	742278	22853	43816	85.5%	92.0%	5.4%
**2010/I**	10799	9140	936276	805104	4473	126765	741693	22313	41098	86.0%	92.3%	5.1%
**2010/II**	10956	9290	958828	827947	4376	126573	768583	21791	37573	86.3%	92.4%	4.5%
**2010/III**	11123	9468	980925	849665	4289	127041	792663	21195	35807	86.6%	92.4%	4.2%
**2010/IV**	11171	9617	989771	865271	3898	120674	808229	22985	34057	87.4%	92.3%	3.9%
**2011/I**	11258	9761	974608	859468	3418	111790	803378	24123	31967	88.2%	92.3%	3.7%
**2011/II**	11333	9870	994656	880602	3347	110776	824465	25470	30667	88.5%	92.3%	3.5%
**2011/III**	11467	10030	1013429	901100	3305	109118	845603	26049	29448	88.9%	92.4%	3.3%
**2011/IV**	11480	10089	1021398	910156	3063	108245	857736	24301	28119	89.1%	92.5%	3.1%
**2012/I**	11588	10196	1014121	906295	3068	104831	858296	21730	26269	89.4%	92.6%	2.9%
**2012/II**	11612	10261	1019125	914177	2916	102114	867192	20862	26123	89.7%	92.6%	2.9%
**2012/III**	11651	10338	1032814	929619	2661	100626	883234	21460	24925	90.0%	92.4%	2.7%
**2012/IV**	11574	10334	1023954	925347	2423	96245	882817	20421	22109	90.4%	92.5%	2.4%
**2013/I**	11428	10229	980141	890397	2109	87707	852262	18571	19564	90.8%	92.7%	2.2%
**2013/II**	11092	9978	960764	876969	1508	82345	843148	16594	17227	91.3%	92.8%	2.0%
**2103/III**	10760	9725	879002	804520	1199	73313	775500	14296	14724	91.5%	92.7%	1.8%
**2013/IV**	8358	7610	363973	331301	621	31848	317585	7227	6489	91.0%	92.5%	2.0%

Determined patient numbers, observation time and proportions of treated patients as well as TCM use and treatment interruptions in the ClinSurv HIV cohort.

**Figure 3:**
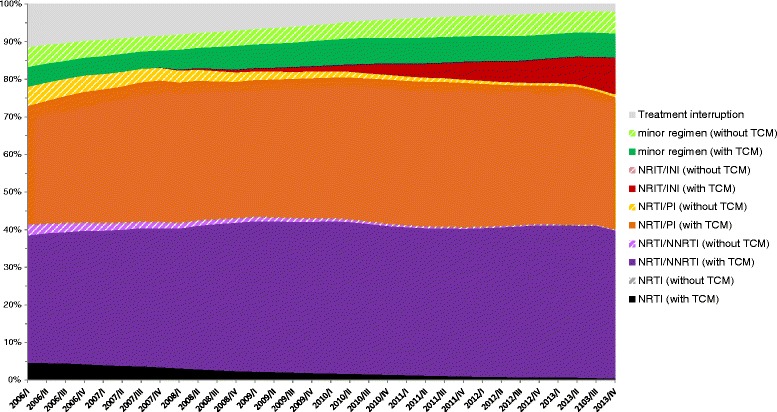
**Composition of ART regimens of patients in the ClinSurv HIV cohort.**

**Figure 4:**
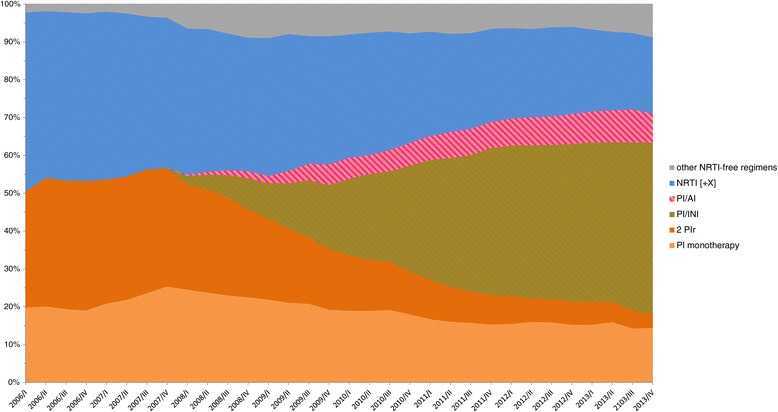
**Composition of minor non-TCM containing ART regimens of patients in the ClinSurv HIV cohort.**

The exact composition of ART regimens of the CSH is shown in Figure 3. The proportion of non-TCM regimen among NRTI/NNRTI and NRTI/PI dramatically decreased over the study period. Non-TCM regimens were most frequently observed among minor regimen which was the only group with a slight increase of only 1% over the study period. The differentiated analyses of the group minor regimens without TCM showed that over the study period, the proportion of any non-TCM-NRTI containing regimen (TCM-NRTI [+X]) as well as the proportion of regimens consisting of two PIs or PI monotherapy decreased, whereas the dual combinations PI/AI, PI/II and other NRTI-free regimens increased continuously from 2007 to 2013 (Figure 4).

### Antiretroviral prescription data (APD)

The number of TCM-containing prescriptions increased from 1,778,070 prescribed DDDs in 2006/I to 3,838,620 prescribed DDDs in 2013/IV.

Taking into account the number of days per quarter led to the number of patients receiving SHI covered TCM containing ART. We observed a systematic seasonal variation, with a disproportionately high number of prescriptions in the last quarter of each year. The number of patients receiving SHI covered TCM-containing ART increased from 19,756 persons in 2006/I to 41,724 persons in 2013/IV. The proportion of persons covered by SHI was different in the respective federal states and ranged from approximately 80% to 90%. The weighted proportion of persons covered by SHI used for the calculation was on average 83.7% over the study period (Table 2).

**Table 2:** **German population, SHI coverage and calculated weighted SHI-coverage factor**

**Year/quarter**	**German population**	**Number of people in SHI**	**SHI-coverage nationwide**	**Weighted SHI-coverage factor**
**2006/I**	82314906	70013157	85.1%	83.2%
**2007/I**	82217837	70022112	85.2%	83.5%
**2008/I**	82002356	69952132	85.3%	83.4%
**2009/I**	81802257	69719142	85.2%	84.1%
**2010/I**	81751602	69473638	85.0%	84.3%
**2011/I**	81843743	69311329	84.7%	83.3%
**2012/I**	81843743*	69398840	84.8%	83.9%
**2013/I**	81843743*	69521912	84.9%	84.0%

*updated data for 2012 and 2013 not available yet.

### Determining the number of people living with HIV receiving ART

**Figure 5:**
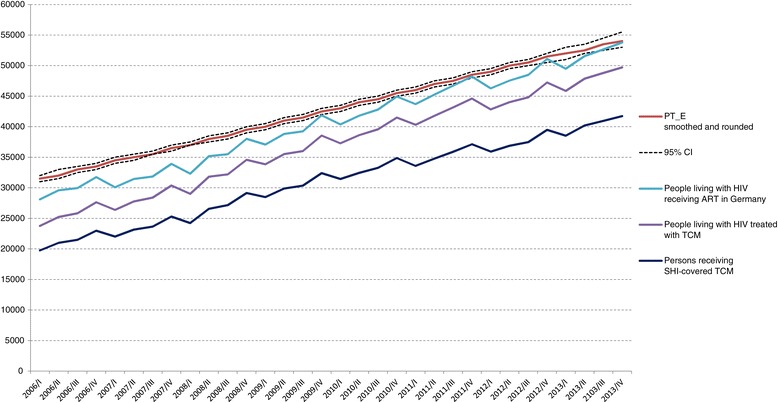
**SHI-covered TCM prescriptions and estimated numbers of HIV infected people with ART experience in Germany, 2006 to 2013.** Step by step estimation of the number of HIV-infected people with ART experience shown as smoothed and rounded numbers, exact numbers are shown in Table 3.

After accounting for patients without SHI by adding 16.3% to the patient numbers derived from APD, the numbers of people living with HIV receiving TCM-containing ART in Germany were 23,751 in 2006/I and increased to 49,719 in 2013/IV. By compensating for regimens not containing TCMs, the number of all people living with HIV receiving ART was estimated at 28,101 in 2006/I and increased continuously to 53,776 in 2013/V. Taking into account those who had interrupted therapy led to the total number of HIV-infected people with ART experience in Germany. Due to the observed seasonal variation, we smoothed the trend by using a negative binomial regression with quadratic time trend. The total number of all HIV-infected people with ART experience in Germany increased from 31,500 (95% CI 31,000-32,000) in the first quarter of 2006 to 54,000 (95% CI 53,000-55,500) individuals by the end of 2013 (Table 3 and Figure 5). The average difference between the number of patients in Germany who had initiated ART and those who had left observation because of emigration or death was estimated to be an average of 2,900 persons per year.

**Table 3:** **Step by step calculated data underlying the estimation of the number of people living with HIV receiving ART in Germany, 2006 to 2013**

**Year/quarter**	**Days per quarter**	**DDDs of TCM from APD**	**Persons receiving SHI-covered TCM**	**Weighted SHI-coverage factor**	**People living with HIV treated with TCM**	**TCMs in the CSH**	**People living with HIV receiving ART in Germany**	**Proportion of interruptions in the CSH**	**HIV-infected people with ART experience in Germany (PT_E)**	**PT_E statistically smoothed**	**95% CI**	**95% CI**	**PT_E smoothed and rounded N (95% CI)**
**2006/I**	90	1778070	19756	83.2%	23751	84.5%	28101	10.9%	31547	31505	30796	32229	31500 (31000-32000)
**2006/II**	91	1910070	20990	83.2%	25222	85.3%	29586	10.2%	32953	32198	31559	32848	32000 (31500-33000)
**2006/III**	92	1975770	21476	83.1%	25824	86.2%	29960	9.8%	33203	32896	32321	33480	33000 (32500-33500)
**2006/IV**	92	2114310	22982	83.1%	27641	87.0%	31757	9.2%	34971	33600	33082	34125	33500 (33000-34000)
**2007/I**	90	1982490	22028	83.5%	26385	87.7%	30092	9.0%	33064	34310	33838	34787	34500 (34000-35000)
**2007/II**	91	2106480	23148	83.3%	27776	88.4%	31434	8.6%	34396	35024	34588	35465	35000 (34500-35500)
**2007/III**	92	2174850	23640	83.3%	28383	89.1%	31844	8.2%	34687	35743	35330	36159	35500 (35500-36000)
**2007/IV**	92	2326950	25293	83.3%	30377	89.5%	33926	7.9%	36841	36467	36066	36872	36500 (36000-37000)
**2008/I**	91	2204460	24225	83.4%	29023	89.8%	32312	7.7%	35009	37195	36794	37600	37000 (37000-37500)
**2008/II**	91	2418270	26574	83.5%	31814	90.4%	35196	7.3%	37964	37926	37516	38339	38000 (37500-38500)
**2008/III**	92	2498580	27158	84.3%	32211	90.7%	35508	7.1%	38226	38661	38237	39089	38500 (38000-39000)
**2008/IV**	92	2680710	29138	84.2%	34578	91.0%	38009	6.8%	40781	39399	38957	39845	39500 (39000-40000)
**2009/I**	90	2562540	28473	84.1%	33844	91.3%	37072	6.4%	39595	40139	39678	40604	40000 (39500-40500)
**2009/II**	91	2719650	29886	84.1%	35529	91.6%	38809	6.1%	41336	40882	40403	41366	41000 (40500-41500)
**2009/III**	92	2792580	30354	84.3%	36015	91.8%	39239	5.8%	41667	41627	41132	42127	41500 (41000-42000)
**2009/IV**	92	2980560	32397	84.0%	38544	92.0%	41876	5.4%	44274	42374	41866	42887	42500 (42000-43000)
**2010/I**	90	2829630	31440	84.3%	37290	92.3%	40385	5.1%	42556	43121	42605	43643	43000 (42500-43500)
**2010/II**	91	2952420	32444	84.0%	38619	92.4%	41794	4.5%	43783	43869	43348	44396	44000 (43500-44500)
**2010/III**	92	3060450	33266	84.1%	39564	92.4%	42800	4.2%	44681	44618	44096	45146	44500 (44000-45000)
**2010/IV**	92	3208470	34875	84.0%	41494	92.3%	44947	3.9%	46790	45367	44847	45892	45500 (45000-46000)
**2011/I**	90	3021690	33574	83.3%	40316	92.3%	43696	3.7%	45388	46115	45599	46636	46000 (45500-46500)
**2011/II**	91	3162900	34757	83.2%	41771	92.3%	45256	3.5%	46888	46862	46349	47379	47000 (46500-47500)
**2011/III**	92	3301830	35889	83.2%	43160	92.4%	46721	3.3%	48301	47607	47095	48124	47500 (47000-48000)
**2011/IV**	92	3414960	37119	83.2%	44619	92.5%	48217	3.1%	49756	48351	47832	48874	48500 (48000-49000)
**2012/I**	91	3268320	35916	83.9%	42827	92.6%	46271	2.9%	47652	49092	48555	49634	49000 (48500-49500)
**2012/II**	91	3356700	36887	83.8%	44007	92.6%	47543	2.9%	48944	49831	49260	50407	50000 (49500-50500)
**2012/III**	92	3447960	37478	83.6%	44816	92.4%	48483	2.7%	49819	50566	49942	51197	50500 (50000-51000)
**2012/IV**	92	3632040	39479	83.6%	47240	92.5%	51089	2.4%	52344	51298	50599	52006	51500 (50500-52000)
**2013/I**	90	3467760	38531	84.0%	45861	92.7%	49478	2.2%	50591	52026	51230	52834	52000 (51000-53000)
**2013/II**	91	3657690	40194	84.0%	47861	92.8%	51555	2.0%	52585	52748	51834	53677	52500 (52000-53500)
**2103/III**	92	3768660	40964	84.0%	48791	92.7%	52657	1.8%	53639	53466	52413	54539	53500 (52500-54500)
**2013/IV**	92	3838620	41724	83.9%	49719	92.5%	53776	2.0%	54849	54178	52967	55416	54000 (53000-55500)

## Discussion

We estimated the number of people living with HIV who received ART based on SHI prescription data and on ART history data from the CSH. An underlying assumption was that the ART regimens and treatment interruptions recorded in the CSH would similarly apply to HIV-infected people outside of the cohort and that the prescription numbers in the APD would be comparable with all people living with HIV in Germany.

In the 2006–2013 observation period, substantial increases were observed for the number of people living with HIV receiving ART and for the number of HIV-infected people with ART experience in Germany. Concomitantly, the use of regimens that included TCMs increased continuously, whereas treatment interruptions in the CSH decreased remarkably.

In an earlier estimation approach by Kollan et al., the calculation was based on the daily drug dosages of all substances. In our opinion, the new approach of calculating the number of individuals based mainly on unambiguous drugs (TCMs in this study) offers a simple and appropriate method that could be further adapted for other investigations.

At the beginning of the observation period, the percentage of CSH regimens that did not include TCMs was 15%, and it decreased by half over time.

In Germany and other industrialised countries with a large number of available antiretroviral drugs, the share of TCMs would need to be taken into account when using this approach to estimate the number of people living with HIV under antiretroviral treatment. However, in countries with fewer antiretroviral drug options, the number of people living with HIV receiving ART could potentially be calculated exclusively using the number of delivered TCMs, which would be a reliable and simple estimation method. Assuming that the proportion of TCM use in Germany will continue to increase, this approach could become even more effective for calculating German estimates.

The total number of all HIV-infected people with ART experience in Germany was estimated to be 31,500 in the first quarter of 2006 and increased continuously to 54,000 individuals by the end of 2013. According to our estimation, the observed study population of the CSH represents more than 20% of all treated patients in Germany. In the CSH all patients who are seen in the centres are automatically included into the cohort without the need for written informed consent. The CSH is therefore the least biased source available and is the largest nationwide cohort of HIV-positive patients. Nonetheless, the CSH in this study is only used to determine the corresponding proportion of non-TCM and treatment interruptions. In our opinion, the demographics do not affect the TCM proportion of those with access to ART. In order to verify this approach with regard to more uncommon ART regimens and first-line subsequent regimens we analysed the composition of regimens of the CSH patients. As shown, the vast majority of ART regimens in the CSH are main regimens which include two or three NRTIs and another drug class such as NNRTIs, PIs, INIs (Figure 3). This applies for first-line therapies as well as for following regimens considering we pooled all data of CSH patients together for the analysis of ART regimens, and therefore regimens after first-line therapy naturally had a greater impact. Non-TCM regimens were most frequently observed within the group minor regimen which was also the only group with a slight increase of only 1% over the study period. Until 2010, within the minor regimen group double or mono PIs and non-TCM-NRTI containing regimens were most frequently observed, and from 2010 to the end of the observation period NRTI-sparing regimens, e.g. PI/AI and PI/INI continuously increased. If the prescribing patterns regarding regimens without TCMs would change in the future then this would have to be considered for our approach. However, this is not the case for the described study period.

It is interesting to note the considerable decline in CSH treatment interruptions. This reflects recent findings showing that there are more risks than benefits from so-called drug holidays [35-37]. In current HIV treatment guidelines, structured treatment interruptions are no longer recommended and are only considered individually under special circumstances [38]. However, currently between 2% of interruption time is apparently an inevitable fact.

In the APD data, we observed a systematic seasonal effect, with the fewest prescriptions at the beginning of each year and the most by the end of the year. We speculate that this effect may be caused by differing patient demand driven by practical considerations with regard to the beginning of the new year (i.e., Christmas holidays, closing of medical offices) and/or prescription co-payments whose reimbursements depend on the annual amounts of all individual co-payments within a calendar year.

Our approach may lead to an overestimation of the number of people receiving continuous ART by patients receiving only short-term ART. This might be relevant in case of discontinuation of therapy early in a quarter or when patients received a PEP.

When a person discontinued therapy before the medication was consumed, we counted that person as someone who was treated, but this person would not get prescriptions in the next quarter, and the overestimation would have been offset in the next billing period.

Representative data regarding the number of PEP prescriptions are rare. Studies regarding PEP are often performed in certain populations with limited significance for the general public. To account for the overestimation resulting from PEP prescriptions, we attempted to determine the number of PEP prescriptions using available studies and sources. We assumed that most PEP prescriptions would come from physicians who were authorised for the special care of patients with HIV/AIDS according to the HIV/AIDS Quality Assurance Agreement (§ 135 para 2 SGB V). According to our findings, the number of PEP prescriptions was estimated to be approximately 2400–2800 per year in Germany [39,40]. Considering that 12 PEP prescriptions are necessary to result in one patient treated per year, an overestimation of approximately 200 to 233 patients in total could have occurred. In terms of the total number of approximately 54,000 people living with HIV receiving ART in Germany, the resulting overestimation would be comparatively small.

On average, the increase in the number of people living with HIV receiving ART was approximately 2,900 persons per year in Germany. This increase should not be confused with the number of persons who initiated therapy, but rather represents the difference between people who initiated ART and those who discontinued treatment because of emigration or death. Thus, the true number of persons who began treatment is probably higher than the observed difference.

The proportion of people covered by PHI differed among the federal states. Those federal states with higher PHI coverage, e.g. City-States, tend to be those with a higher number of prescriptions. We therefore used a weighted SHI-coverage factor based on the data for each federal state and applied it to the antiretroviral prescription data in order to improve the estimates. Using the nationwide SHI-coverage factor would underestimate the total number by 1.6% (N = 650 persons).

With this study, we provide a nationwide estimate and a useful tool for calculating the number of people living with HIV who received ART, those with ART experience and the increase in ART usage between 2006 and 2013 in Germany using the available number of prescriptions and surveillance data from the CSH.

This approach can be useful to estimate the number of people living with HIV and those receiving ART in other countries. Additionally, the described methodology could potentially be used and adapted for other investigations or medications in the future.

### Limitations

The described approach has some limitations. One limitation is an overestimation resulting from the cases that were discussed above. Of those cases, the number of PEP prescriptions is the most uncertain, which could be the main limitation.

Overall, our aim was to estimate the number of treated patients among all persons with access to ART. We do not aim to, and therefore do not, estimate the number of non-treated patients among all people infected with HIV in Germany.

Lamivudine is approved for the treatment of hepatitis B with a dose of 100 mg once daily for persons not infected with HIV. The use of lamivudine with approval for HIV therapy (150 mg and 300 mg) in the treatment of hepatitis B of HIV-negative individuals attributable to economic considerations cannot be excluded. However, the off-label use of HIV-labelled lamivudine would require an alternative dosing regimen by administration on alternating days and/or by dividing the pills, which we consider impractical in reality.

A limitation with regard to applying this approach in the future is that if TCM prescribing patterns, such as the currently discussed dual NRTI-sparing therapies, or other treatment practices significantly change, the impact of a second source (in our case, the CSH) on the estimate would be greater.

## Conclusions

This report describes the first comprehensive approach to estimating the number of people living with HIV who receive ART. The study provides a possible approach for determining the number of people receiving specialised HIV medical care in Germany. This method allows for contrasting the numbers of people living with HIV receiving ART derived from different sources or estimation approaches. This approach can be useful to estimate the number of people living with HIV and those receiving ART in other countries. The described methodology could be used and adapted for different investigations or medications in the future. Non-TCM regimens and CSH treatment interruptions declined notably. Assuming that this trend will continue in the future, the number of people living with HIV receiving ART could be estimated exclusively using TCM-containing prescriptions. In other settings with fewer available antiretroviral drugs, the estimation would be even more robust.

It is also of interest to note trends in antiretroviral therapy with regard to NRTI-free regimens. In this context, the relevance of data from cohort studies remains very high for observing and assessing such developments.
